# Synergistic antitumor interaction between valproic acid and capecitabine in breast cancer

**DOI:** 10.1186/2051-1426-3-S1-P4

**Published:** 2015-08-14

**Authors:** Manuela Terranova-Barberio, Elena DI Gennaro, Maria Serena Roca, Alfredo Budillon

**Affiliations:** 1 IRCCS National Cancer Institute G. Pascale, Napoli, Italy

## 

Capecitabine, commonly used in different settings for metastatic breast cancer, is an inactive prodrug that take advantage of the tumor elevated levels of thymidine phosphorylase (TP), a key enzyme for its conversion to 5-florouracil. Potentiation of anticancer activity of capecitabine is required to improve its therapeutic index.

We demonstrated that histone deacetylase inhibitors (HDACi), including the anti-epileptic valproic acid (VPA), induced dose and time-dependent upregulation of TP transcript and protein in breast cancer cells but not in non-tumorigenic MCF-10A cell line. By using siRNA or isoform specific HDACi we demonstrated that HDAC-3 is the main isoform whose inhibition is involved in TP modulation. Combined treatment of HDACi, including VPA, and capecitabine resulted in synergistic/additive antiproliferative and proapoptotic effects in all cell lines tested. TP knockdown experiments demonstrated the crucial role of TP modulation in the synergism observed. The synergistic antitumor effect between VPA and capecitabine was also demonstrated in vivo in a breast cancer xenograft model, but not in xenografts from TP-knocked cells, confirming in vitro data. Overall, this study suggests that the combination of an HDACi, such as valproic acid, and capecitabine, is an innovative antitumour strategy and warrants further clinical evaluation for the treatment of metastatic breast cancer.

